# Effects of different microbial agents on bedding treatment of ectopic fermentation of buffalo manure

**DOI:** 10.3389/fmicb.2022.1080650

**Published:** 2022-12-22

**Authors:** Kaifeng Niu, Chen Chao, Xinxin Zhang, Zhigao An, Jiayan Zhou, Liguo Yang

**Affiliations:** ^1^Key Laboratory of Animal Genetics, Breeding and Reproduction, Ministry of Education, College of Animal Science and Technology, Huazhong Agricultural University, Wuhan, China; ^2^International Joint Research Centre for Animal Genetics, Breeding and Reproduction (IJRCAGBR), Huazhong Agricultural University, Wuhan, China; ^3^Hubei Province’s Engineering Research Center in Buffalo Breeding and Products, Wuhan, China

**Keywords:** buffalo manure, ectopic fermentation, bedding material, microbial agents, microbial bacteria distribution

## Abstract

**Introduction:**

The rapid development of the farming industry has increased the amount of manure produced by livestock and poultry, causing increasingly prominent environmental pollution problems. In recent years, due to the increase in conventional bedding material costs, an increasing number of farmers choose to use harmless recycled manure as bedding. Manure bedding treatment of farms can not only solve the problem of manure pollution, but also resource utilization of manure and cost savings.

**Methods:**

This study compared the effects of five microbial agents (Microbial agents A, B, C, E, F) on buffalo manure bedding treatment by testing the temperature, moisture content, pH, microbial bacteria distribution of buffalo manure ectopic fermentation, and screened the lowest cost and most effective agent. The changes of microbial bacteria distribution in different periods of bedding treatment were also detected.

**Results:**

Agent A was eliminated because of poor fermentation effect and low fermentation temperature, which could not achieve the effect of harmless treatment. The other four agents of bacteria achieved a harmless effect, but the bedding treatment effect of agent F was significantly better than agent E, B, and C. In terms of the cost of agents: the cost of agent F required for fermenting 100m^³^ buffalo manure was the lowest, 1000yuan, followed by E (1200yuan), C (1750yuan), and B (1980yuan). In the process of ectopic fermentation bedding treatment of buffalo manure, Firmicutes, Bacteroidetes, Proteobacteria, Actinobacteria, and Chloroflexi were the major bacteria used. The process was divided into three periods; the heating period - high temperature period - cooling period, the high temperature period could reach more than 75°C, and a large number of pathogenic bacteria and harmful bacteria, and other miscellaneous bacteria in the pile were degraded, their species diversity was reduced, and the structure of bacterial flora had significant differences in different treatment periods. In conclusion, this study has provided a guide for the resource utilization of manure in cattle farms and the reduction of manure pollution to the environment.

## 1. Introduction

With the rapid development of the farming industry, the amount of manure produced by livestock and poultry has significantly increased, resulting in increasingly prominent environmental pollution problems (Wei et al., 2020). According to statistics, the total amount of livestock and poultry manure produced in China reached 3.8 billion tons in 2020, including about 1.6 billion tons of bovine manure, and the resource utilization rate is <50% (Li, 2021). Environmental pollution and economic pressure of livestock manure have become major problems that every farm urgently needs to solve (Wang et al., 2020). Due to the increased cost of traditional bedding material, more and more farmers are opting to use recycled manure bedding (Rowbotham and Ruegg, 2016). By treating farm manure with environmentally sound bedding not only can the problem of manure pollution be solved, but also the manure can be resourcefully used (Leso et al., 2020).

There are currently three main approaches to the environmentally friendly treatment of bovine manure for use as bedding material: Firstly, the solid–liquid separation direct utilization model (Fournel et al., 2019a). The manure from cattle farms is collected in the collection tank and then treated by solid–liquid separation, and the separated liquid can be made into organic fertilizer, biogas fermentation, etc. (Pedersen et al., 2020), and some farmers use the residual solid manure residue directly as bedding material after simple drying (Leach et al., 2015); or the solid manure residue is directly used as bedding material through mixing and extrusion (Cole and Hogan, 2016). The operation is simplified, low cost, and short cycle time (Husfeldt et al., 2012), but the bedding material is of poor quality and not harmlessly treated, and the bedding material contains a large number of parasitic eggs and pathogenic bacteria with poor safety (Wu et al., 2010). Secondly, the anaerobic fermentation bedding production model (Tasnim et al., 2017). The manure from cattle farms is collected in a fermenter with the addition of bacterium for anaerobic fermentation (biogas fermentation; Ozbayram et al., 2018), and after the completion of fermentation, the solid digestate is separated out through solid–liquid separation and can be used as bedding after simple treatment (Zhang et al., 2020). This method is simple, low cost, and short cycle time (Zhang et al., 2018), but the most effective results are at 30°C–45°C for medium temperature anaerobic fermentation, incomplete harmlessness (Díaz et al., 2016), and high gas production efficiency, low solids content, and relatively low output of recovered bovine manure solids (Wang et al., 2017). Thirdly, the aerobic fermentation bedding production model (Fournel et al., 2019b). The manure from cattle farms is collected to the treatment area, and the moisture content of the manure is adjusted to about 60% by solid–liquid separation equipment or by adding auxiliary materials (grain hulls, wood chips, etc.; Duan et al., 2015). Bedding material is then generated by adding aerobic fermentation bacteria and corresponding treatment (He et al., 2016). It can be classified as natural pile fermentation (Niu et al., 2022), strip stack aerobic fermentation (Han et al., 2021), tank aerobic fermentation (Hanajima et al., 2011) and drum aerobic fermentation (Chinakwe et al., 2019a,b). This method has a thorough innocuous treatment, high biosafety, and high quality bedding, but high human and material costs and long fermentation cycles (Cole and Hogan, 2016).

The development of an inexpensive, simple, and efficient microbial agent has far-reaching application prospects. This study aimed to compare the fermentation effect of different microbial agents on buffalo manure bedding treatment and the change of microbial species to screen out the optimal microbial agent. To reduce the environmental pollution of manure in cattle farms and improve the resource utilization of waste.

## 2. Materials and methods

This study was conducted from October 2018 to February 2019 in Jingmen City, Hubei Province, China at HUBEI PRIME CATTLE HUSBANDRY CO. LTD.

### 2.1. The experimental microbial agents

A: 1 generation of self-developed microbial agent.

Product formation: Bacillus subtilis, Bacillus licheniformis, Lactobacillus, Saccharomycetes. etc. CFU ≥ 5.0 * 10^8^/g.

B: Fujian Luodong Biotechnology Co., LTD microbial agent.

Product formation: Bacillus subtilis, Fat-soluble bacillus, Beer yeast powder, Defatted rice bran. Etc. CFU ≥ 5.0 * 10^8^/g.

C: Henan Nongfukang Biological Technology Co., LTD microbial agent.

Product formation: Bacillus licheniformis, Bacillus subtilis, Lactobacillus, Candida utilis. Etc. CFU ≥ 1.0 * 10^7^CFU/g.

E: Henan Hebi Renyuan Biotechnology Development Co., LTD microbial agent.

Product formation: Bacillus subtilis, Bacillus licheniformis, Lactobacillus, Cellulase, Protease, Amylase. etc. CFU ≥ 1.0 * 10^8^/g.

F: 2 generation of self-developed microbial agent.

Product formation: Bacillus subtilis, Lactobacillus, Saccharomycetes, Aspergillus oryzae, Cellulase, Hemicellulase, Ligninase, Pectinase. etc. CFU ≥1.0 * 10^10^/g.

### 2.2. Experimental method

**Figure 1:**
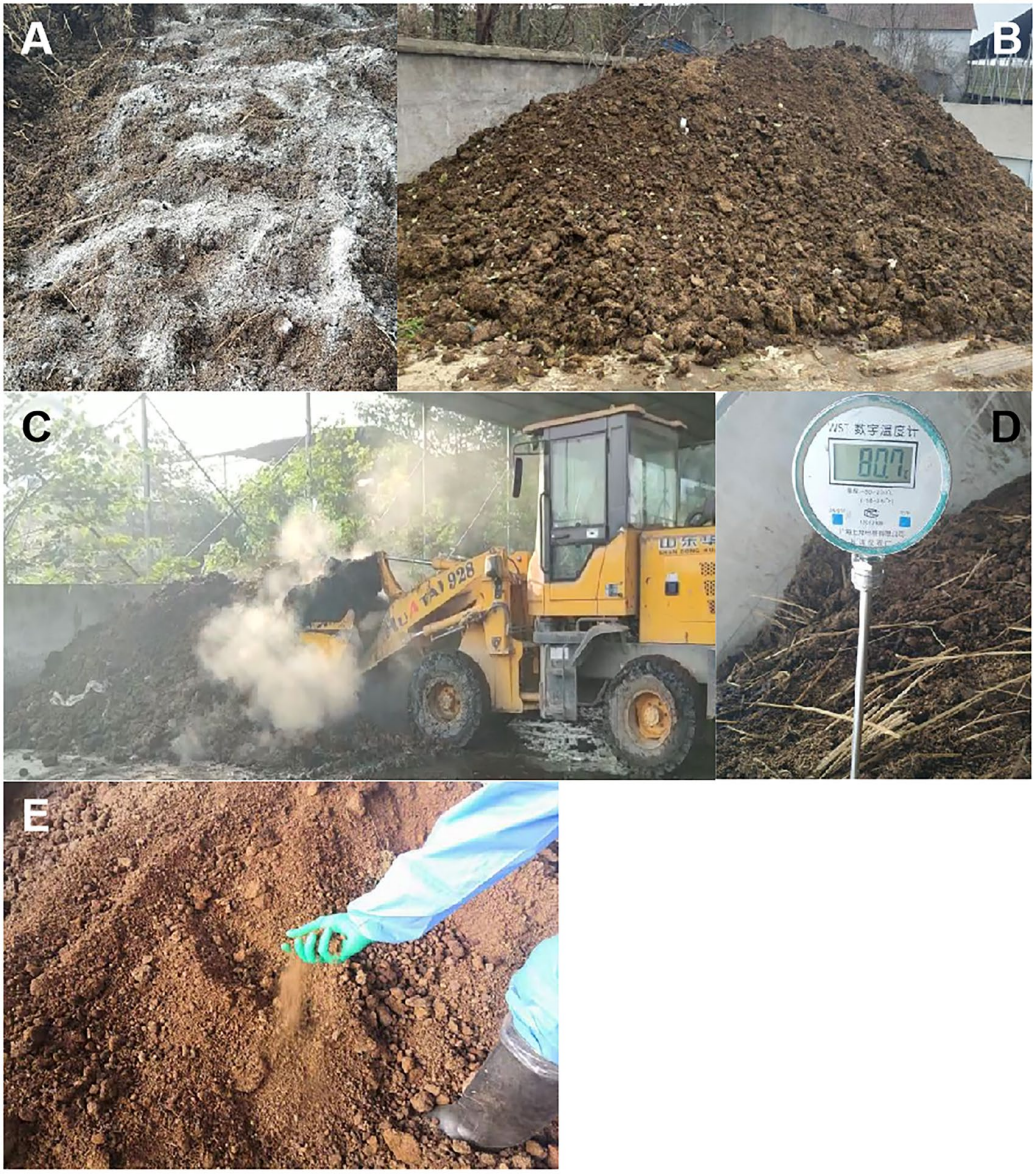
The production process of harmless fermentation bedding material of buffalo manure. **(A)** Adjust the moisture content of buffalo manure and add microbial agent. **(B)** Turning over evenly and pile up. **(C)** Turning the pile regularly. **(D)** Check the temperature daily. **(E)** Fermented manure bedding material.

The process of using buffalo manure as raw material and harmless ectopic fermentation treatment as bedding material is shown in Figure 1. Firstly, fresh buffalo manure and rice chaff were collected and mixed by use of a forkliftto adjust the moisture content of the mixture to about 60% in a pile. Then, the microbial agents were evenly sprinkled and mixed to build a strip chopped heap with length 8 m, width 6 m and height 1.5 m. The temperature of the pile was detected with a handheld digital display thermometer every day. When the temperature of the pile reaches 55°C, turn the pile with forklift every 4 days thereafter.

#### 2.2.1. Experiment 1

Four ectopic fermentation piles were established according to the above method, in which piles A, B and C were added with agent A, B and C. Pile D was the control group without any agent added. The experimental period was October 25–December 5, 2018, with an ambient temperature of 2.4–18.7°C and a period of 41 days.

#### 2.2.2. Experiment 2

Four ectopic fermentation piles were established according to the above method, in which three piles B, E and F were added with B agent, E agent and F agent, respectively. Pile D was the control group without any agent added. The experiment was conducted from December 20, 2018 to February 2, 2019, with an ambient temperature of −2.7 to 10.2°C and an experimental period of 45 days.

### 2.3. Testing index

Apparent physical characteristics: The piles were observed daily for color, volume, odor, texture, and other properties.

Temperature: Each pile was selected as a temperature measurement point around and on top of the pile, and the temperature of the surface layer of 20 cm, the middle layer of 45 cm and the deep layer of 70 cm was measured by digital thermometer at 8:00 a.m. and 15:00 p.m. every day. Then the average value was taken as the actual temperature of the surface layer, middle layer and deep layer of the pile, and the ambient temperature at that time was recorded.

Moisture content: The washed aluminum trays were dried in a constant temperature drying oven at 105°C until constant weight and recorded as W1. 50 g of fermented manure samples were taken, laid flat in the trays, and weighed and recorded as W2, put in a drying oven at 105°C for 12 h until constant weight, and then cooled for 30 min and weighed and recorded as W3. Each pile was weighed and recorded as W3 according to “Z” five-point sampling, the moisture content of the five samples were tested and the average value was taken.

Moisture content = (W2-W3)/(W2-W1) × 100%.

pH: The pH value was measured by taking fermented manure samples, putting them into a centrifuge tube, adding distilled water, centrifuging them, and taking the supernatant after standing. Each pile was sampled at five points in the shape of “Z,” and the average value was taken after testing the moisture content of five samples.

Microbial bacteria distribution: The samples were taken on day 1 (initial period), day 7 (heating period), day 20 (high temperature period), and day 45 (completion period) of the buffalo manure ectopic fermentation bedding treatment, and the samples were counted in groups A, B, C and D. Each group was sampled at five points in a “Z” shape, and five samples were taken for 16S sequencing. Five samples were taken for 16S sequencing.

### 2.4. Statistical analysis

Statistical analysis was performed by SPSS software (SPSS v. 31, SPSS Inc.; Chicago, IL, United States). Significance analysis based on the production performance, behavioral indicators, animal welfare indicators, and were conducted by one-way ANOVA in SPSS. *p* < 0.05 was used to indicate a significant difference.

## 3. Results

### 3.1. Results of experiment 1

#### 3.1.1. Apparent characteristics

Throughout the fermentation process, the four groups of manure underwent great changes in color, volume, odor, texture, and other traits. At the beginning of the experiment, the buffalo manure in the four groups was black in color and had a severe irritating odor. The moisture content was high, viscous, and could be formed into a ball with a firm grip, with water oozing out. There was black cloudy liquid oozing out from the bottom of the piles.

In the early period of the experiment (heating period), the manure of group A had a warm feeling when touched by hand, while group D did not heat up obviously. Neither group A nor D had obvious white smoke coming out, there was a pungent odor, the moisture content of buffalo manure was high, viscous, and could be clumped up when held tightly by hand. The temperature of the pile of groups B and C increased, white smoke came out, there was a burning feeling when touched by hand. There was a pungent odor, and a large amount of white mycelium grew out of buffalo manure. The moisture content decreased, and the pile could still be clumped up with a tight hand, and no liquid exuded.

In the middle period of the experiment (high temperature period), the manure of group A had a warm feeling when touched by hand, while the pile of group D did not heat up obviously. The manure of group A and D had no obvious white smoke coming out when turning, there was a pungent odor. The manure had high moisture content and viscous, and it could be clumped up when held tightly by hand. The temperature of the pile of groups B and C was high, a large amount of white smoke came out when turning, there was a burning feeling when touched by hand. The ammonia odor of buffalo manure gradually disappeared, and the white mycelium disappeared. The color began to change to dark brown, the volume of the pile was obviously reduced, the manure was dry, and the sense of granularity and fluffiness became more and more obvious.

At the end of the experiment (completion period), the volume of piles in groups A and D basically did not decrease, the color did not change significantly, and there was little difference from before the ectopic fermentation treatment. The manure has a pungent ammonia odor, humidity, sticky, and could be clumped up into lumps when held tightly by the hand. There was a large amount of slatted lumpy buffalo manure. The volume of piles in groups B and C was reduced by about a quarter. The color of manure from black to dark brown, no pungent odor, there was an earthy smell. There were no large clumps of slatted buffalo manure, and the manure was loose, dry, and fluffy, with obvious granularity when gripped by hand.

#### 3.1.2. Temperature

**Figure 2:**
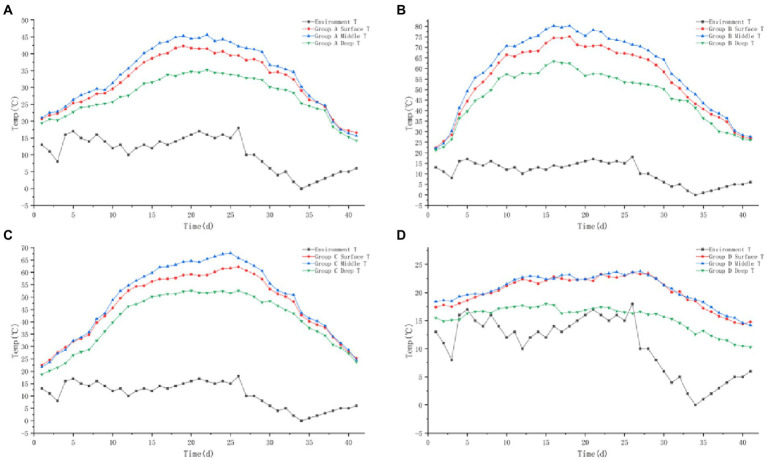
Variation trend of surface, middle, deep temperature and ambient temperature of pile A (microbial agent A - **A**), B (microbial agent B - **B**), C (microbial agent C - **C**) and D (control - **D**).

The temperature trends of the four groups A, B, C, and D are shown in Figures 2A–D: the temperature of the piles in groups A, B, and C all showed three periods of the heating period – high temperature period – cooling period. The temperature of the middle layer of the pile was the highest in the high temperature period, which was 3–5°C higher than the surface layer of the pile and about 15°C higher than the deep layer of the pile. The temperature of the pile in control group D was only slightly higher than the ambient temperature and failed to reach the high temperature period.

The maximum temperature of the surface layer of pile A was 42.3°C, the middle layer was 45.6°C, the deep layer was 35.2°C. The maximum temperature of the middle layer of the pile failed to reach 50°C, and the buffalo manure failed to achieve the effect of pollution-free treatment. The maximum temperature of the surface layer of pile B was 75.2°C, the middle layer was 80.3°C, the deep layer was 63.4°C. The medium layer of the pile took 6 days to heat up from the initial temperature to 50°C, and the duration over 50°C was 28 days; it took 8 days to heat up to 60°C, and the duration over 60°C was 23 days; it took 10 days to heat up to 70°C, and the duration over 70°C was 18 days. The maximum temperature of the surface layer of group C pile was 62.2°C, the middle layer was 67.8°C and the deep layer was 52.6°C. It took 11 days to heat up the middle layer of the pile from the initial temperature to 50°C, and 23 days to last longer than 50°C; it took 16 days to heat up to 60°C, and 14 days to last longer than 60°C; the maximum temperature of the pile failed to reach 70°C. The B and C groups of buffalo manure completely achieved the harmlessness effect. The maximum temperature of the pile was 23.6°C in the surface layer, 23.8°C in the middle layer and 18.0°C in the deep layer of the control group D. The temperature of the pile was only slightly higher than the ambient temperature. It failed to achieve harmlessness (Table 1).

**Table 1:** Effects of different agents on fermentation temperature of buffalo manure.

Item	D	A	B	C	SEM	Value of *p*
Time required to heat up to 50°C (d)	Fall flat	Fall flat	6	11	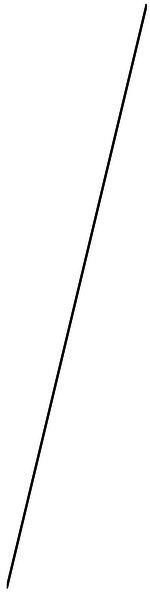	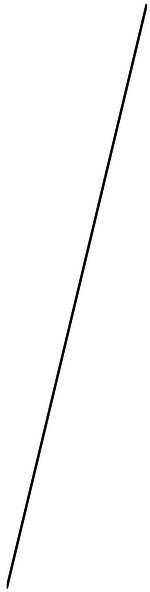
Time required to heat up to 60°C (d)			8	16		
Time required to heat up to 70°C (d)			10	Fall flat		
Duration exceeding 50°C (d)			28	23		
Duration exceeding 60°C (d)			23	14		
Duration exceeding 70°C (d)			18	Fall flat		
Maximum surface T (°C)	23.6	42.3	75.2	62.2		
Maximum middle T (°C)	23.8	45.6	80.3	67.8		
Maximum deep T (°C)	18.0	35.2	63.4	52.6		
Mean T during the high temperature period (°C)	Fall flat	Fall flat	71.18	62.83	1.32	<0.01

Buffalo manure in group A and group D failed to reach the high temperature period above 50°C, the average temperature of high temperature period in group B was 71.18°C, and the average temperature of high temperature period in group C was 62.83°C, the difference was significant (*p* < 0.05).

#### 3.1.3. Moisture content

**Figure 3:**
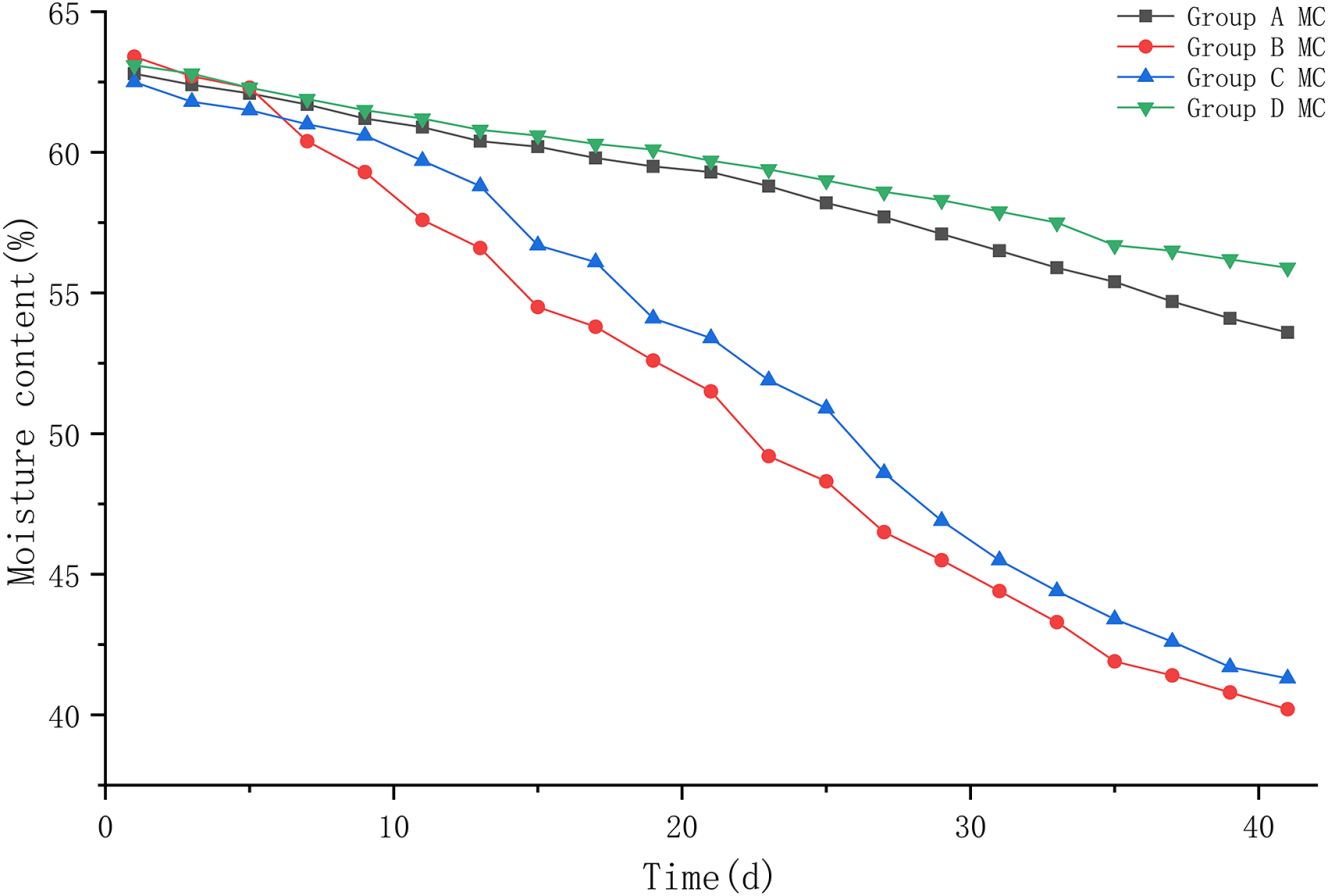
The moisture content trend of pile A (microbial agent A), B (microbial agent B), C (microbial agent C) and D (control).

The initial moisture contents of the piles in groups A, B, C, and D were 62.8%, 63.4%, 62.5%, and 63.1%, respectively. With the treatment of ectopic fermentation bedding, the moisture content of the piles all showed a decreasing trend, and the moisture contents of piles A, B, C, and D were 53.6%, 40.2%, 41.6%, and 55.9%, respectively, at the end of the innocuous treatment (Figure 3). Comparing the changes in moisture content of the four piles, it was found that pile B changed the fastest, followed by pile C, and piles A and D changed more slowly with significant differences (*p* < 0.05).

#### 3.1.4. pH

**Figure 4:**
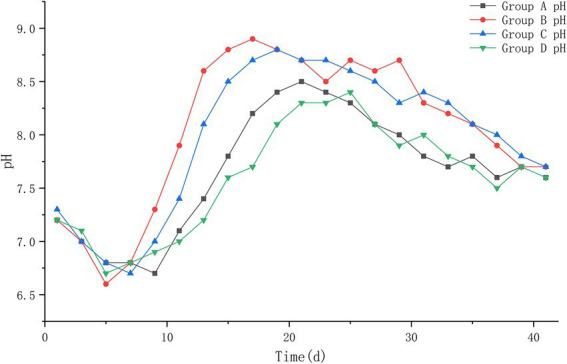
The pH trend of pile A (microbial agent A), B (microbial agent B), C (microbial agent C) and D (control).

The results of this experiment showed that: At the beginning of the experiment, the pH values of the four groups were 7.2, 7.2, 7.3, and 7.2, and during the heating period of the piles, the pH values decreased to about 6.6; during the high temperature period, the pH values of group B and C increased until the highest value of 8.9–9.0; the pH values of group A and D increased to the highest value of 8.4–8.5; during the cooling period, the pH values of the piles began to decrease slowly and leveled off. At the end of the experiment, the pH values of all four piles were roughly stable between 7.6 and 7.7, and a slightly alkaline environment was basically maintained in the piles. The pH of each pile was compared at different periods after treatment, and it was found that group B had the fastest change, decreasing to 6.6 on the 5th day of treatment, and then began to rise rapidly to 8.8 on the 15th day after treatment; group C was the next. Group A and D changed more slowly and with less variation (Figure 4).

### 3.2. Results of experiment 2

#### 3.2.1. Apparent characteristics

**Figure 5:**
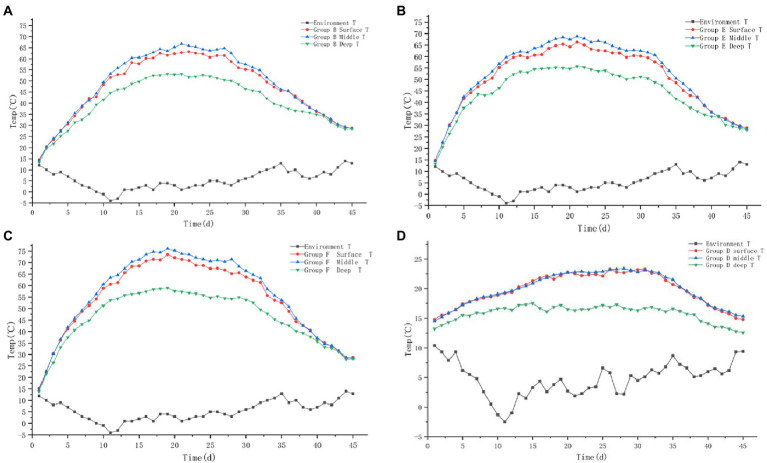
Variation trend of surface, middle, deep temperature and ambient temperature of pile B (microbial agent B - **A**), E (microbial agent E - **B**), F (microbial agent F - **C**) and D (control - **D**).

The color, volume, odor, fluffiness, and other traits of the piles in the four groups changed greatly during the whole fermentation process. The process of changing the apparent physical characteristics of the piles in group D was similar to that in the control group of experiment 1; groups B, E, and F were similar to that in group B of experiment 1 (Figure 5).

#### 3.2.2. Temperature

The temperature trends of the four groups B, E, F, and D are shown in Figures 5A-D. The trend of temperature change in experimental group (B, E, F) and control group D was similar to that in experiment 1.

The maximum temperature of the surface layer of Group B pile was 63.2°C, the middle layer was 66.7°C, and the deep layer was 53.2°C. It took 11 days for the middle layer of the pile to heat up from the initial temperature to 50°C, and 23 days to exceed the duration of 50°C; 14 days to heat up to 60°C, and 15 days to exceed the duration of 60°C; it failed to reach 70°C. The maximum temperature of the surface layer of the pile in group E was 66.3°C, the middle layer was 68.8°C, the deep layer was 55.7°C. It took 8 days for the middle layer of the pile to heat up from the initial temperature to 50°C, and 28 days for the duration over 50°C; 12 days to heat up to 60°C, and 21 days for the duration over 60°C; failed to reach 70°C. The maximum temperature of the surface layer of the pile in group F was 73.5°C, the middle layer was 76.1°C, the deep layer was 58.9°C. It took 8 days for the middle layer of the pile to heat up from the initial temperature to 50°C and 29 days for the duration over 50°C; 10 days to heat up to 60°C and 23 days for the duration over 60°C; 14 days to heat up to 70°C and 15 days for the duration over 70°C. The three groups of buffalo manure completely achieved the harmlessness effect. The maximum temperature of the pile was 23.3°C in the surface layer, 23.4°C in the middle layer and 17.5°C in the deep layer of the control group D. The temperature of the pile was only slightly higher than the ambient temperature. It failed to achieve harmlessness (Table 2).

**Table 2:** Effects of different agents on fermentation temperature of buffalo manure.

Group	D	B	E	F	SEM	Value of *p*
Time required to heat up to 50°C (d)	Fall flat	11	8	8	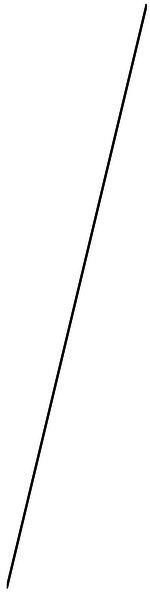	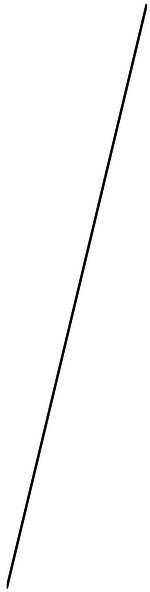
Time required to heat up to 60°C (d)		14	12	12		
Time required to heat up to 70°C (d)		Fall flat	Fall flat	14		
Duration exceeding 50°C (d)	Fall flat	23	28	29		
Duration exceeding 60°C (d)		15	21	23		
Duration exceeding 70°C (d)		Fall flat	Fall flat	15		
Maximum surface T (°C)	23.3	63.2	66.3	73.5		
Maximum middle T (°C)	23.4	66.7	68.8	76.1		
Maximum deep T (°C)	17.5	53.2	55.7	58.9		
Mean T during the high temperature period (°C)	Fall flat	62.09	63.82	68.43	1.33	<0.01

The pile body temperature of control group D was only slightly higher than the ambient temperature and failed to reach the high temperature period. The average temperature of the high temperature period of group B was 62.09°C, the average temperature of the high temperature period of group E was 63.82°C, and the average temperature of the high temperature period of group F was 68.43°C, with significant differences (*p* < 0.01).

#### 3.2.3. Moisture content

**Figure 6:**
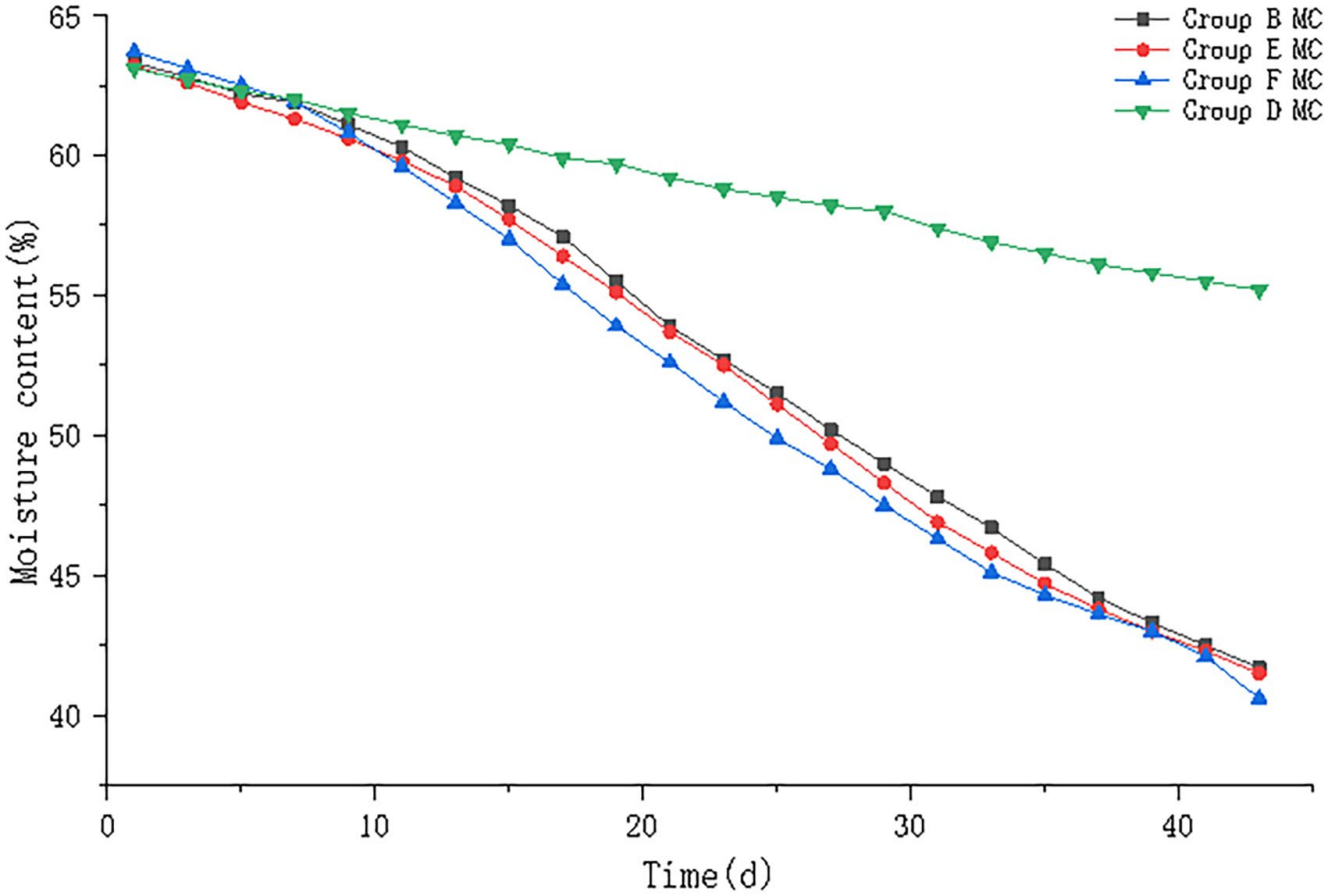
The moisture content trend of pile B (microbial agent B), E (microbial agent E), F (microbial agent F) and D (control).

The results of pile moisture content are shown in Figure 6. The moisture content of all four groups of piles showed a decreasing trend, in which the moisture content of piles in control group D decreased from 63.1% to 55.2%; the moisture content of piles in group B decreased from 63.3% to 41.7%; the moisture content of piles in group E decreased from 63.2% to 41.5%, and the moisture content of piles in group F decreased from 63.7% to 40.6%. B, E, F groups were not significantly different (*p* > 0.05).

#### 3.2.4. pH

**Figure 7:**
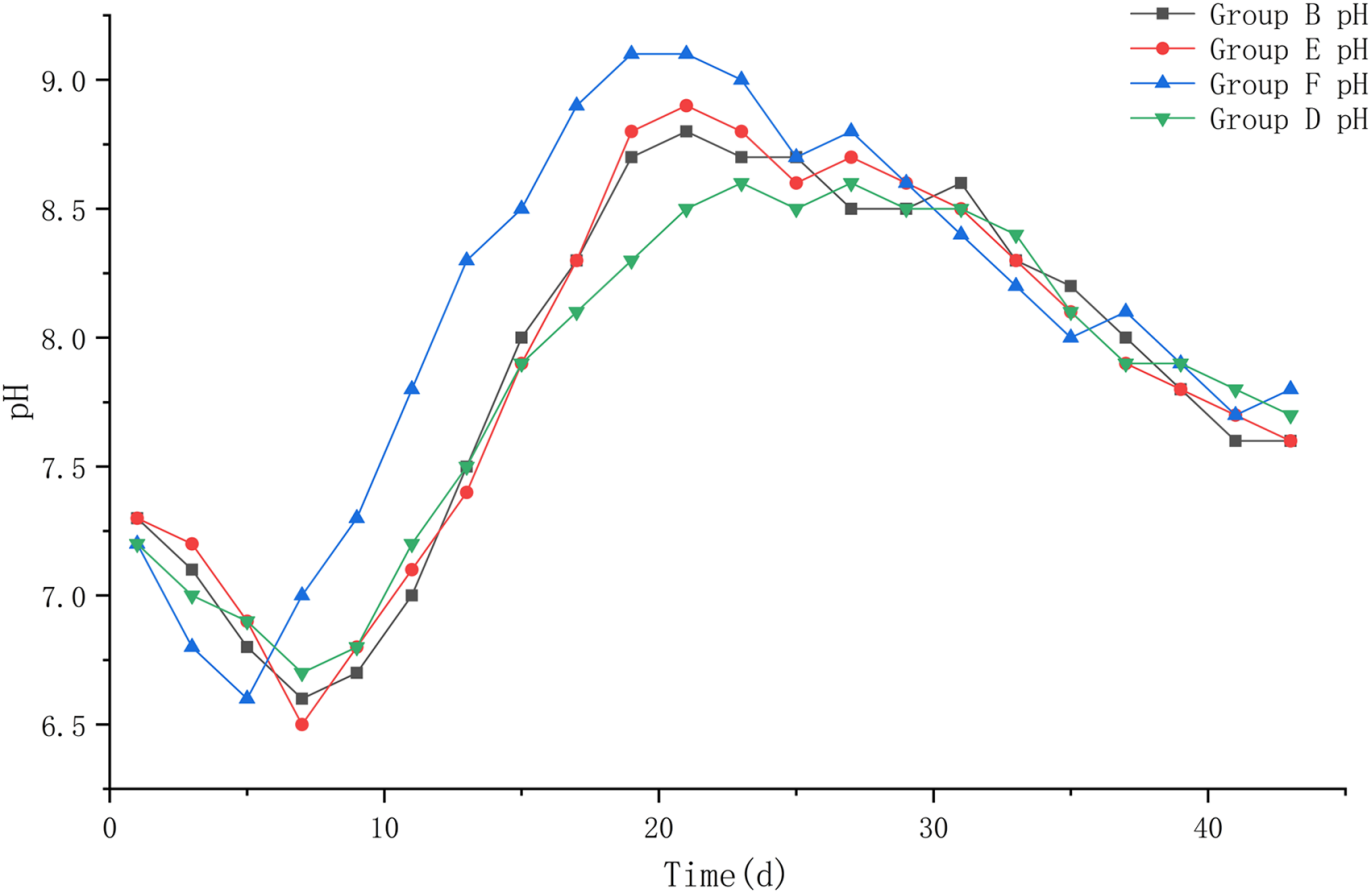
The pH trend of pile B (microbial agent B), E (microbial agent E), F (microbial agent F) and D (control).

The pH values of the four groups at the beginning of the experiment were 7.3, 7.3, 7.2, and 7.2. The pH values decreased to about 6.6 during the heating period of the piles; during the high temperature period, the pH values showed an increase until the highest value of about 9.0; during the cooling period, the pH values of the piles began to decrease slowly and leveled off. At the later period of the experiment, the pH values of all four piles were roughly stabilized at about 7.7, and a slightly alkaline environment was basically maintained in the piles. Compared with the pH of each pile at different periods after treatment, it was found that the pH of pile F changed the fastest and started to increase rapidly after the 5th day after treatment, and reached the highest value on the 17th day after treatment; piles B and E followed. Pile D changed more slowly and with less variation (Figure 7).

#### 3.2.5. Microbial bacteria distribution

**Figure 8:**
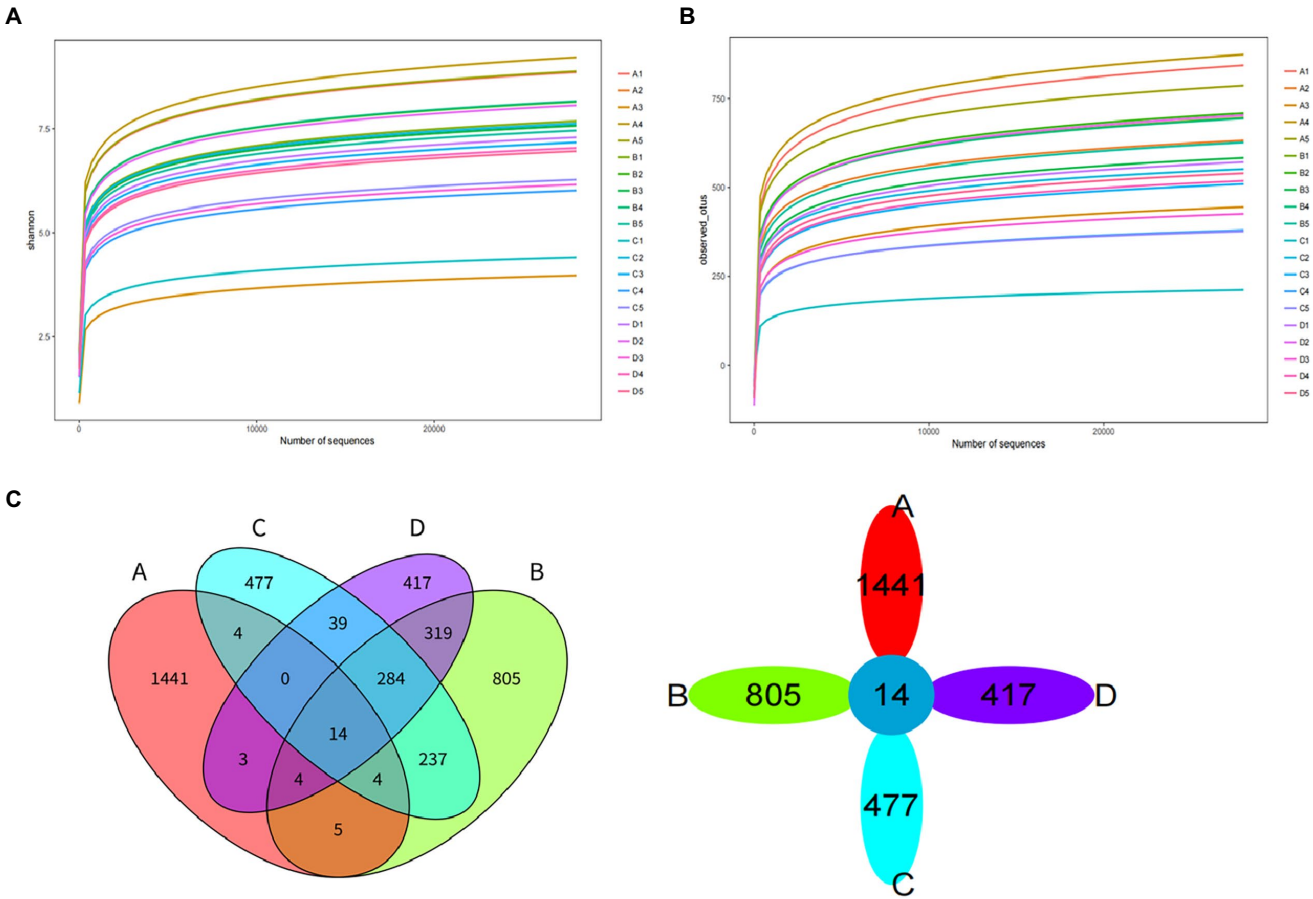
Diversity index dilution curve of group F. **(A)** Shannon diversity index dilution curve. **(B)** Observed-OTUs diversity index dilution curve. **(C)** Venn diagram of OTU of pile F.

The dilution curve of Shannon diversity index dilution curve for the 20 samples in group F is shown in Figure 8A, and the dilution curve of Observed-OTUs is shown in Figure 8B. This is used to reflect the microbial diversity of each sample.

The clustering of Operational Taxonomic Units (OTUs) for 20 samples from Group F ectopic fermented buffalo manure at a 97.0% similarity level is shown in Figure 8C. There were 14 OTUs in four groups of samples. 1441 OTUs were unique to group A (initial period), 805 OTUs were unique to group B (heating period), 477 OTUs were unique to group C (high temperature period), and 417 OTUs were unique to group D (completion period).

**Figure 9:**
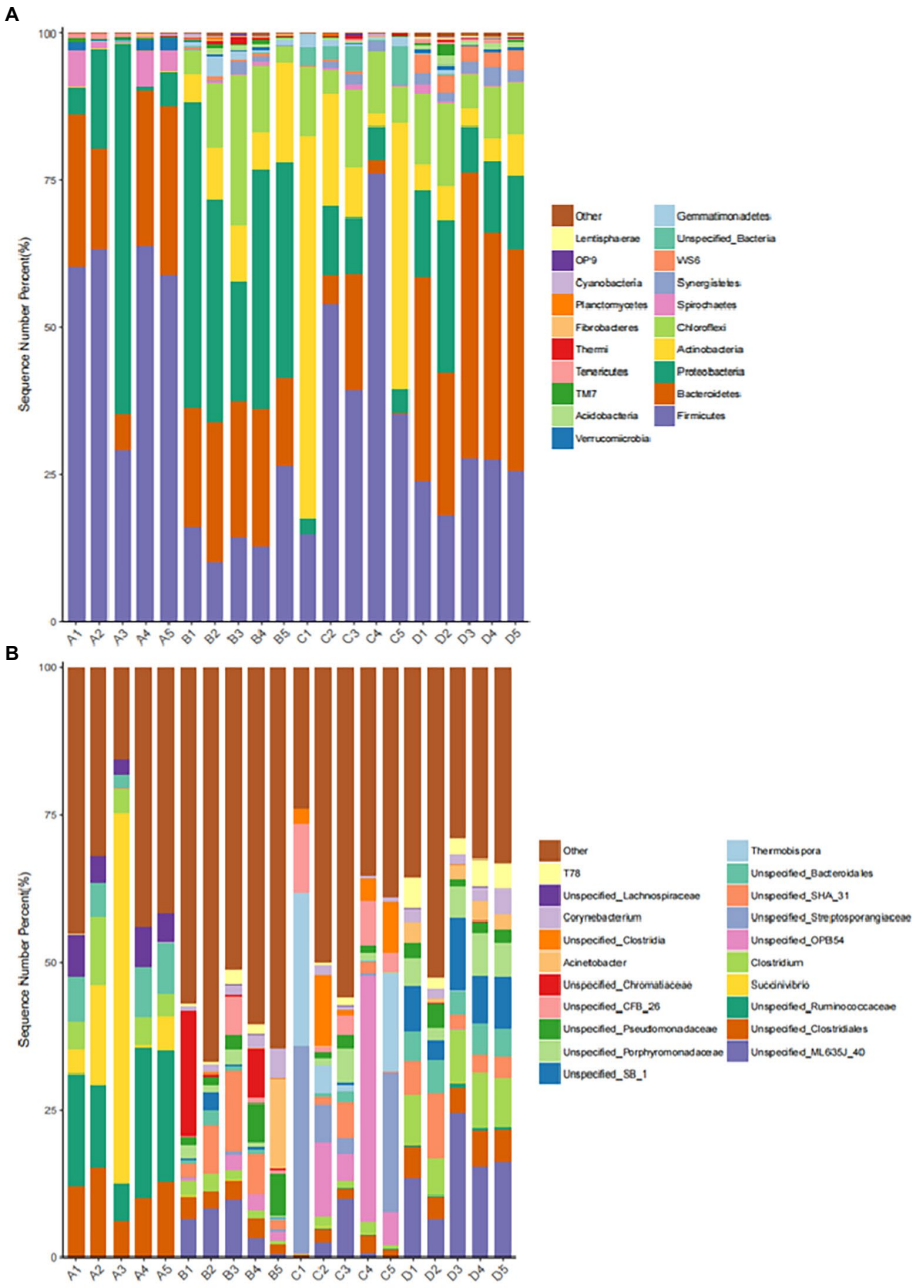
Distribution of dominant microbial bacteria. **(A)** Distribution of dominant microbial bacteria at phylum level. **(B)** Distribution of dominant microbial bacteria at genes level.

The abundance of dominant bacteria in different periods of fermented manure in group F in this experiment: the level of dominant phylum in group A (initial period): (Firmicutes 55.01%), (Bacteroides 20.90%), (Proteobacteria 18.22%) were the main groups. The level of dominant phylum in group B (heating period): (Proteobacteria 37.49%), (Bacteroides 21.13%), (Firmicutes 15.98%) were the main groups. The level of dominant phylum in group C (high temperature period): (Firmicutes 43.88%), (Actinobacteria 28.04%) were the main groups. (Proteobacteria 6.70%), (Bacteroides 5.50%) decreased in abundance. The level of dominant phylum in group D (completion period): (Bacteroides 36.78%), (Firmicutes 24.53%), (Proteobacteria 14.73%) were the main groups (Figure 9A).

The abundance of dominant bacteria (genus level) in different periods of fermented manure in group F in this experiment: Group A (initial period): (Succinivibrio 17.13%), (Ruminococcaceae 15.82%), (Clostridium 13.27%), (Bacteroidaceae 6.54%), (Lachnospiraceae 5.22%). Group B (heating period): (Bacteroidaceae 11.91%), (Anaerolineae 9.34%), (Clostridium 8.86%), (Chromatium 5.42%), (Pseudomonadaceae 4.77%). Group C (high temperature period): (Clostridium 14.24%), (Streptosporangiaceae 12.92%), (Thermobispora 9.7%), (Anaerolineae-CFB-26 5.36%). Group D (completion phase) dominant: (Bacteroidaceae-ML-635J-40, Bacteroidaceae-SB-1 28.12%), (Clostridium 17.54%), (Anaerolineae-SHA-31 8.92%), (Porphyromonadaceae 5.04%). (Figure 9B).

**Figure 10:**
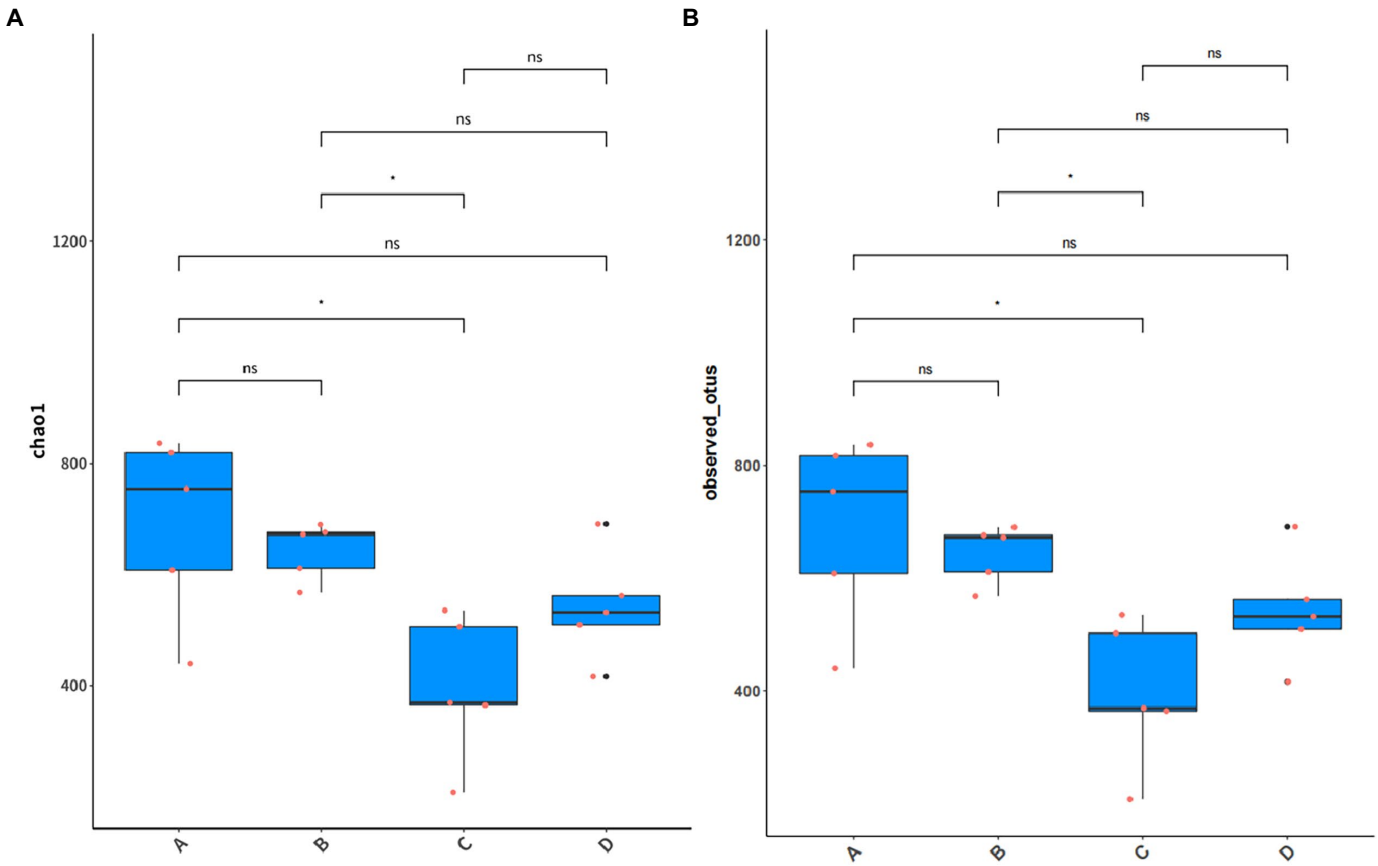
Alpha diversity analysis of manure in group F. **(A)** Chao 1 index. **(B)** Observed-OTUs index.

The results of Alpha diversity analysis of samples from different periods of the buffalo manure in group F are shown in Figure 10. Chao 1 index responded to species abundance and Observed-OTUs index responded to microbial diversity. The difference between group A and C was significant, between group B and C was significant (*p* < 0.05), and between the rest of the groups was not significant (*p* > 0.05).

**Figure 11:**
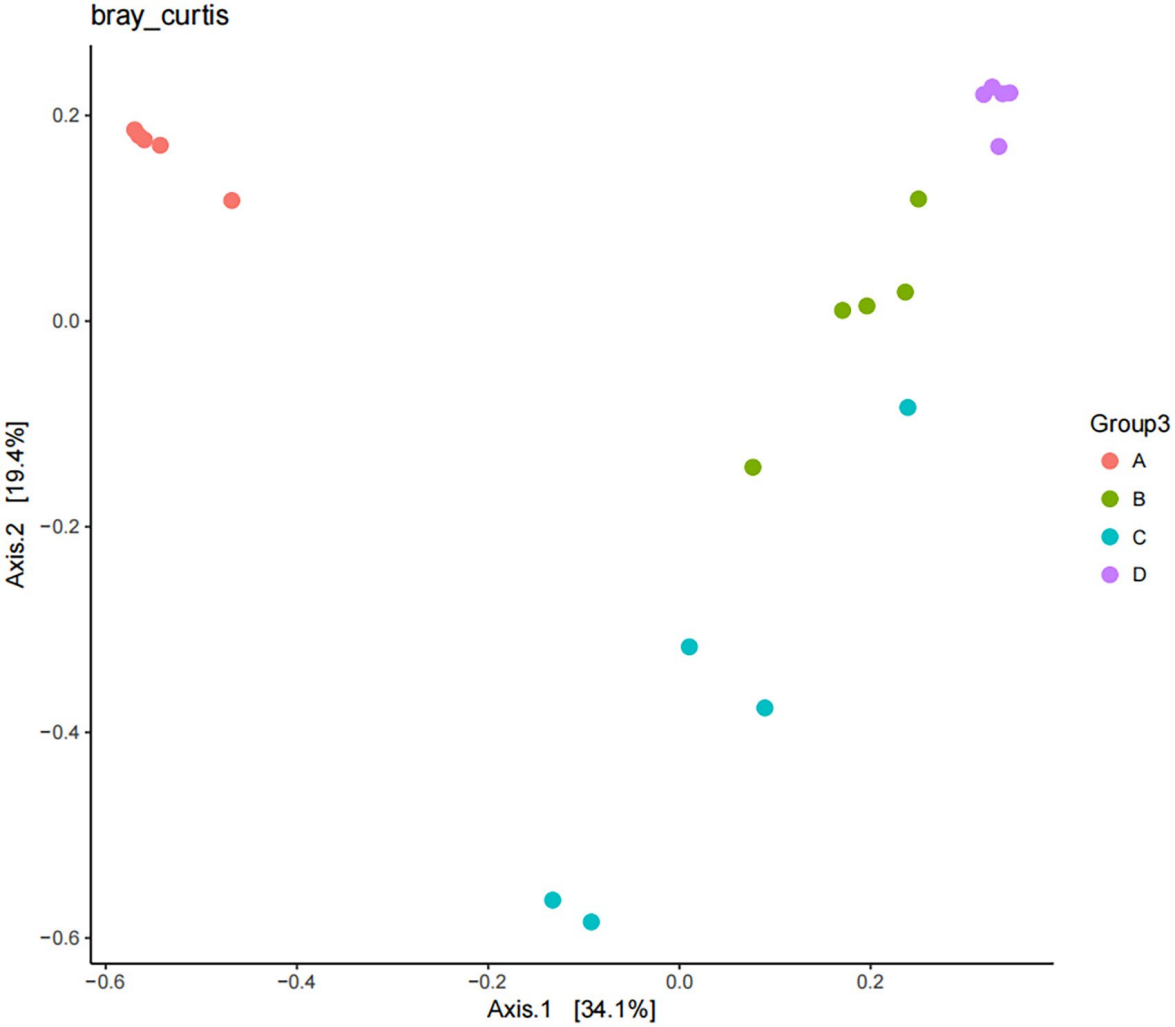
Beta diversity analysis of manure in group F.

The results of Beta diversity analysis of sample from different periods of the buffalo manure in group F are shown in Figure 11. Based on Bray Curtis to perform PCoA (Principal coordinates analysis) analysis, the closer the distance of the samples in the graph, the more similar the species composition structure of the samples is indicated. The first principal component contributed 34.10% to the sample variance and the second principal component contributed 19.40% to the sample variance, adding up to a contribution of 53.50%. The manure samples were closer together in the same period and the differences were smaller. The manure samples were farther apart in in different periods with larger differences. This indicates that there is a statistical difference between the samples of manure in different periods during the ectopic fermentation treatment.

The comparison of colony structure between groups based on the Beta diversity index is shown in Table 3. Significant differences (*p* < 0.01) in colony structure were observed between the four period samples of group F piles.

**Table 3:** Comparison of bacterial community structure between groups based on Beta diversity index.

Group 1	Group 2	Sample size	Value of *p*
A	B	10	<0.01
	C	10	<0.01
	D	10	<0.01
B	C	10	<0.01
	D	10	<0.01
C	D	10	<0.01

### 3.3. Costing

The cost analysis of different agents is shown in Table 4, where agent A failed to meet the requirement of environmentally friendly treatment and was eliminated. Agent B, C, E, and F could treat buffalo manure harmless, whereas agent B had the highest cost, fermenting 100 m^3^ of manure at the cost of 1,981 yuan. Agent F had the lowest cost of 1,000 yuan.

**Table 4:** Cost of different agents.

Microbial agent	Application amount (g/m^3^)	Dosage of microbial agent for fermentation of 100 m^3^ bovine manure (kg)	Price (yuan/kg)	Cost of microbial agent for fermentation of 100 m^3^ bovine manure (yuan)	Ferment effect
A	125	12.5	80	1,000	Fail
B	165	16.5	120	1,980	Excellent
C	40	4.0	350	1,750	Fine
E	200	20.0	60	1,200	Excellent
F	125	12.5	80	1,000	Excellent

## 4. Discussion

### 4.1. Physicochemical indexes

#### 4.1.1. Apparent characteristics

Buffalo manure underwent great changes in traits such as color, volume, odor, and fluffiness throughout the bedding process (Kwang et al., 2005). At the beginning of the experiment, the pile of manure was in the form of black clumps with slight water oozing from the bottom, a heavy odor, and some flies could be seen around the pile. The pile was heated up to 55°C or above and regularly turned over, and the manure was thoroughly turned over with a shovel and spread out to dry for 1–2 h before stacking. Adequate turning of the pile allows manure to be in full contact with air and aerobic bacteria to be fully effective (Ge et al., 2020). The high moisture content of manure in the pre-experimental period will have a large amount of slabby clumpy buffalo manure. The temperature of the pile rose, a large amount of white smoke was produced when the pile was turned, a large number of thermophilic bacteria multiplied and expanded and took effect, and a large number of white mycelium grew in the manure. A strong irritating odor was mixed, probably due to the decomposition of the organic matter in the raw material by the associated bacteria and proteases, which led to the production of amino acids, which then underwent deamination to produce large amounts of NH3 (Bello et al., 2020). At the later stage of the experiment, after sufficient turning of the pile, the pile temperature began to decrease, there was a small amount of white smoke, the ammonia odor gradually disappeared, the white mycelium in buffalo manure disappeared, the color began to change to dark brown, the moisture content decreased, the slate-clumped manure was broken up and presented loose granularity, the volume of the pile decreased significantly, and the granularity and fluffiness of the pile became more and more obvious. This is similar to the trait changes in the composting process of bovine and pig manure (Kwang et al., 2005). The total volume of the pile was reduced by about one-fourth at the end of the experiment, and the color of bovine manure changed from the original black to dark brown with no pungent odor and an earthy smell, probably due to the action of bacteria such as actinomycetes at a later stage, which produces substances such as earthy pigments (Debasis et al., 2022). There was no slabby clumpy dung, dry and fluffy, with obvious granularity.

#### 4.1.2. Temperature

The temperature index is the most important indicator in the treatment of bovine manure with ectopic fermentation bedding (Miyatake and Iwabuchi, 2006). Temperature in the process of manure ectopic fermentation bedding treatment is the most important factor affecting the microbial activity in the pile, and it is also the most intuitive and sensitive indicator reflecting the success of ectopic fermentation bedding (Chinakwe et al., 2019a,b). According to the requirements of China’s “Hygienic Standard for Harmless Manure (GB7959-87),” bovine manure can basically meet the requirements of harmlessness if the fermentation temperature can exceed 50°C and stay for 5–7 days during the harmless treatment process. In this study, we used this criterion to judge whether buffalo manure was harmless or not. The pile temperature of group A and two control groups did not reach above 50°C, so it failed to meet the requirements of harmless treatment. While the other groups of buffalo manure can reach more than 50°C or even 60°C and stay for more than 7 days, so it meets the requirements of harmless treatment.

The experiment compared the treatment effect of 5 kinds of microbial agents: A, B, C, E, and F. Where the control group had no microbial agent added, the temperature was only slightly higher than the ambient temperature and did not reach the high temperature period. The rest of the groups all experienced three temperature change periods: heating period – high temperature period – cooling period, which were in line with the general rule of change of the temperature of ectopic fermentation. The experimental results showed that the agent A could not render the manure harmless and was eliminated. The remaining four microbial agents were able to harmlessly treat manure as bedding material. Among them, F bacteria had the best fermentation effect, followed by E bacteria, B bacteria, and C bacteria. The mean temperature of the buffalo manure pile during the high temperature period was significantly different (*p* < 0.01). The treatment effect of agent B in experiment 1 was better than that in experiment 2. It is speculated that this was because, during the cold winter season, the ambient temperature of manure bedding treatment was −2.7 to 10.2°C, and the heat was easily dissipated, which had an effect on the effect of manure bedding treatment.

#### 4.1.3. Moisture content

The moisture content of the pile has an important influence on the treatment of buffalo manure ectopic fermentation bedding. The moisture content of fresh buffalo manure can reach about 80%, with high humidity and high viscosity, if fermentation is carried out directly, the pile will have poor permeability and will not meet the oxygen content required for microbial growth and reproduction, and microorganisms will not be able to play their role. Moreover, the high moisture content and high viscosity result in fluid bovine manure, which cannot be piled up or turned (Ge et al., 2022). Therefore, it is necessary to artificially adjust the moisture content of bovine manure, and this experiment was conducted to control the moisture content of manure by adding chaff. Adequate turning of the pile during the fermentation process can ensure that the material is in full contact with air and the aerobic bacteria, and it can promote water evaporation under high temperature conditions (Ge et al., 2020).

The moisture content of the piles in this experiment all showed a decreasing trend, and the decrease in moisture content was correlated with the fermentation temperature. The higher the fermentation temperature the more obvious the decrease in moisture content of the piles. In experiment 1, the moisture content of groups A and D decreased from about 63% to 55%, and the bacteria in group A was eliminated; the moisture content of the rest of the experimental groups decreased from about 63% to about 40%. The decreasing trend of the moisture content of the piles with the addition of different microbial agents was not significantly different (*p* > 0.05), but the moisture content of the buffalo manure bedding materials all reached a sufficiently dry level. In general, there is a tendency for the moisture content of the piles to decrease due to natural evaporation, but the addition of microbial agents allows the water in the bovine manure to be more fully utilized, resulting in an accelerated decrease in the moisture content of the piles (Ghanney et al., 2021).

#### 4.1.4. pH

Various microbial activities require a suitable acid and alkaline environment during the treatment of bovine manure with ectopic fermentation bedding. Studies have indicated that the appropriate pH for bovine manure during fermentation is neutral or weakly alkaline (Sadeli et al., 2022). In the present study, the pH of all groups varied a little between 6.5 and 9.0, with a trend of rapid decline followed by an increase and then a decrease to a flat and slightly alkaline environment. The initial bovine manure pH was roughly between 7.0 and 7.3, and decreased rapidly in the early stage of pile fermentation, presumably due to the rapid multiplication of non-methanogenic bacteria at the beginning, which produced large amounts of ethanoic acid and CO_2_. Dark brown acidic cloudy liquid also oozed out from the bottom of the pile at the beginning, and the pH decreased (Dai et al., 2016). After turning the pile, the bovine manure entered the high temperature period and the pH began to rise, reaching a maximum of about 8.7–9.0. The explanation is that ammonification bacteria began to multiply and act in large numbers, rapidly decomposing proteins and other substances in the raw material to produce amino acids, and then generating a large amount of NH_3_ to neutralize some acids through deamination, while methanogenic bacteria converted ethanoic acid, CO_2_ and H_2_ into CH_4_, and the pH of bovine manure gradually increased. This explained the irritating ammonia odor during the turning process in the early stage of the experiment (Bello et al., 2020). At the later stage of the experiment, with the participation of a large number of microbial agents in nitrification, the nitrogenous organic matter was used up and the irritating odor largely disappeared, and the pH slowly decreased and stabilized. This is consistent with the findings in the studies of Kim et al. (2012) and Yamane (2016). The use of pH indicators to track the environment within the pile during bovine manure fermentation can provide effective insight into the utilization of organic matter by microorganisms within the pile so that the turning conditions can be adjusted in time for smooth bovine manure fermentation (Sadeli et al., 2022).

#### 4.1.5. Microbial bacteria distribution of fermentation bedding In different periods

The distribution of the microbial bacteria flora during the treatment of bovine manure with ectopic fermentation bedding had significant differences in different treatment periods. The main bacteria in the buffalo manure bedding process in this study included firmicutes, bacteroidetes, proteobacteria, actinobacteria, and chloroflexi. Similar to the distribution of the main bacteria in pig manure (Wei et al., 2022) and bovine manure during aerobic composting (Wang et al., 2018; Meng et al., 2019). During the process of ectopic fermentation of bovine manure, it underwent a heating period – high temperature period – cooling period, and the high temperature period could reach more than 75°C, and a large number of harmful bacteria were killed (Chinakwe et al., 2019a,b).

In the initial period, firmicutes (55.01%), bacteroidetes (20.90%), and proteobacteria (18.22%) were the dominant bacterial groups. At the genus level, succinivibrio (17.13%) and ruminococcaceae (15.82%) were the dominant bacteria, both of which were isolated from the rumen and intestine of cattle and sheep, etc. The initial period had not yet started fermentation and the pile was mainly a mixture of fresh buffalo manure and hulls, so they became the dominant bacteria (Guo et al., 2021).

During the heating period, firmicutes (15.98%) decreased, proteobacteria (37.49%) and bacteroidetes (21.13%) became the dominant bacteria, both of which mainly played the role of degrading organic matter. Proteobacteria and bacteroidetes also played the same role during the heating period in the buffalo manure composting process (Duan et al., 2021). At the genus level, bacteroides (11.91%) and anaerolineae (9.34%) became the dominant bacteria, and pseudomonadaceae (4.77%) and chromatium (5.42%) increased in abundance. The bacteria can all participate in organic matter metabolism and can degrade proteins, carbohydrates, and other organic matter (Wang et al., 2016). In addition, clostridium (8.86%) increased in abundance. Bacillus clostridiales belongs to the firmicutes – clostridiaceae, with sporulation, and is a very unique species in nature, mainly fo und in soil, human and animal intestines and spoilage (Xu et al., 2022). As the pile temperature started to the abundance of *Clostridium* spp. began to increase as the temperature of the pile began to rise.

During the high temperature period, the pile fermentation temperature could reach above 75°C and thermophiles proliferated rapidly becoming the dominant bacterial flora. At this time, the relative abundance of proteobacteria (6.70%) and bacteroidetes (5.50%) decreased, while firmicutes (43.88%) and actinobacteria (28.04%) were the dominant bacterial flora in the high temperature stage, which is consistent with the results of Meng et al. (2019). These thermophiles can decompose structurally complex organic matter (hemicellulose, cellulose, and lignin, etc.). Firmicutes and actinobacteria endospores are tolerant to high temperatures and can survive and proliferate as dominant bacteria during the high temperature phase (Wang et al., 2016). At the genus level, the dominant bacteria during this period were clostridium (14.24%), streptosporangiaceae (12.92%), and thermobispora (9.7%) with increased abundance. Mesophiles decreased in abundance and thermophiles proliferated to become dominant, with most of the genera having budding spores, heat tolerant, and thermophilic (Zhong et al., 2020).

In the completion period, the abundance of mesophiles such as firmicutes (24.53%) decreased. Bacteroidetes (36.78%) and proteobacteria (14.73%) increased and regained dominance to continue decomposing the difficult organic matter. At the genus level, bacteroidaceae (28.12%), clostridium (17.54%), and anaerolineae (8.92%) became the dominant bacteria. In this study, firmicutes was the dominant bacteria in all treatment periods. Firmicutes mainly originated from the intestinal tract of livestock and had the function of decomposing and utilizing a variety of carbohydrates, which indicates that the degradation and metabolism of carbohydrates are ongoing during the treatment of bovine manure by ectopic fermentation bedding (Wan et al., 2021). Anaerolineae was the dominant bacterium in both the high temperature and completion period. It belongs to chloroflexi – anaerolineae. Anaerolineae can be involved in denitrification and play an important role in the treatment of sludge, waste sewage, and livestock manure pollutants (Mcilroy et al., 2017).

In summary, the distribution of the microbial bacteria flora of buffalo manure in the process of ectopic fermentation bedding treatment changes accordingly at different times. The species diversity of the samples decreases, and a large number of pathogenic and harmful bacteria and other miscellaneous bacteria in the pile are degraded.

## 5. Conclusion

This study compared the effect of five different microbial agents on the treatment of buffalo manure bedding and the changes in microbial flora distribution during the treatment process to screen out the optimal microbial agent. Among them, Agent A failed to meet the requirements of harmless treatment and was eliminated. Other microbial agents could ferment buffalo manure into bedding material, and agent F had the lowest cost and the best treatment effect. This study provides a basis for resource utilization of farming manure, reducing the cost of bedding and reducing the pollution of the environment by manure in cattle farms.

## Data availability statement

The raw data supporting the conclusions of this article will be made available by the authors, without undue reservation.

## Author contributions

KN and LY contributed to conception and design of the study. CC organized the database. XZ performed the statistical analysis. KN wrote the first draft of the manuscript. ZA and JZ wrote sections of the manuscript. All authors contributed to the manuscript revision, read, and approved the submitted version.

## Funding

This work was supported by the Modern Agro-industry Technology Research System: CARS-36.

## Conflict of interest

The authors declare that the research was conducted in the absence of any commercial or financial relationships that could be construed as a potential conflict of interest.

## Publisher’s note

All claims expressed in this article are solely those of the authors and do not necessarily represent those of their affiliated organizations, or those of the publisher, the editors and the reviewers. Any product that may be evaluated in this article, or claim that may be made by its manufacturer, is not guaranteed or endorsed by the publisher.
